# Cannabis health knowledge and risk perceptions among Canadian youth and young adults

**DOI:** 10.1186/s12954-020-00397-w

**Published:** 2020-08-03

**Authors:** Cesar Leos-Toro, Geoffrey T. Fong, Samantha B. Meyer, David Hammond

**Affiliations:** 1 School of Public Health & Health Systems, University of Waterloo, 200 University Ave. W., Waterloo, ON N2L 3G1 Canada; 2 Department of Psychology, University of Waterloo, 200 University Ave. W, Waterloo, ON N2L 3G1 Canada; 3 Ontario Institute for Cancer Research, MaRS Centre, 661 University Avenue, Suite 510, Toronto, ON M5G 0A3 Canada

**Keywords:** Cannabis literacy, Cannabis health knowledge, Cannabis risk perceptions, Synthetic cannabis, Public health education

## Abstract

**Abstract:**

**Background:**

Although recreational cannabis is now legal in Canada, little empirical evidence exists regarding young Canadians’ cannabis literacy, cannabis-related risk perceptions, and risk of different forms of cannabis or the effect that public health education may have on these perceptions. The present study sought to address these knowledge gaps to examine health knowledge and risk perceptions associated with cannabis use.

**Methods:**

An online survey was conducted with a national sample (*N* = 870) of Canadians aged 16 to 30 years in October 2017 using a commercial panel. The study examined young Canadians’ awareness of negative health effects related to cannabis, evaluation of known risks, and risk perceptions of different forms of administration.

**Results:**

Most respondents were aware of a cannabis-related physical health effect (78.0%). Approximately one-third reported having been exposed to public health messaging about cannabis; digital media was reported most frequently. Compared to never users, ever users were less likely to report general likelihood of addiction (*p <* 0.001) and harm to mental health (*p* < 0.001). Approximately one-quarter of past 3-month cannabis users reported they were at least “a little” addicted. Respondents who reported using a particular form of cannabis self-administration (e.g., edibles, smokables) were less likely to perceive harm than those who did not use each form (*p* < 0.001).

**Conclusions:**

The current study is among the first to measure the knowledge and perceptions of risks of Canadian youth about cannabis. The study, conducted in the time immediately preceding legalization, may serve as a reference point for future studies examining changes in cannabis knowledge and risk perceptions. This will be important in addressing the need for monitoring and enhancing public awareness of the impact and potential harms of this newly legalized substance.

## Background

The *Cannabis Act*, which came into force in October 2018, outlines a legal framework for controlling the production, distribution, and possession of cannabis in Canada [1] . Two of its primary goals are to prevent youth from accessing cannabis and to protect public health and safety by allowing safe and legal access of nonmedical cannabis to adults. In Canada, the typical age of initiation of cannabis use is 14 years, with prevalence peaking among young adults aged 20 to 24 years [2–5]. According to the 2017 Canadian Cannabis Survey, past-year use of cannabis among Canadians aged 16 to 24 years was double that of Canadians older than 24 [6]. Cannabis use is higher among certain population subgroups such as young people who report poor school performance, early use of tobacco, alcohol, and other drugs, those fraternizing with drug-using peers, those experiencing a difficult family environment, those who report higher cannabis accessibility and availability of cannabis, and those who are high on sensation-seeking [7, 8].

Youth who initiate cannabis at an earlier age may be at greater risk of negative health and social outcomes than those that delay initiation [9]. Young Canadians report a variety of reasons for using cannabis, including identity formation, as a tool for social cohesion, boredom, relief of social anxiety, perceived acceptability, and lower perceived risk of harm than other substances [10–14]. However, early cannabis use has been associated with greater impairments in memory, cognition, academic performance, heightened risk for cannabis dependence, and other negative health endpoints than those who delay or refrain from use [15–17]. Other general health effects include the exacerbation of mental health disorders, respiratory symptoms, increased risk of motor vehicle accidents, and lower birthweight of infants whose mothers smoked cannabis [17]. The majority of health consequences related to first-hand cannabis use occur among individuals who are high frequency and high intensity users [9, 18, 19].

Most young Canadians report that regular cannabis use has health risks. For example, the 2016-17 Canadian Student Tobacco, Alcohol and Drugs Survey (CSTADS), a nationally representative survey of youth, found that while 9% of youth reported that there was “no risk” of harm associated with smoking cannabis on a regular basis, approximately half (54%) thought that smoking cannabis on a regular basis posed “great risk” [2]. Data suggest that most young Canadians are aware of specific potential consequences associated with use; however, they do not appear to have an understanding of practical issues such as how long impairment might persist [12]. Among the general population, three-quarters of young Canadians (75%) believe that cannabis affects driving, and approximately 1 in 5 (19%) report that it does not affect driving at all [6]. Young Canadians report taking solace in the fact that cannabis affects everyone differently. Focus groups have found that young people are indifferent to the consequences associated with use and selectively decide the harms that use may elicit based on personal experience or anecdotes from their peers [12]. Indeed, many young people perceive cannabis as “natural,” a “soft drug,” or “not really a drug at all” [11, 12]. Approximately 6 in 10 Canadians report that cannabis is more helpful than harmful to their mental and physical health [13].

To date, most studies have assessed risk perceptions for cannabis use in general, simply probing about whether individuals believe that cannabis carries a level or risk. Few studies have examined risk perceptions for different types of cannabis products (e.g. edibles, concentrates) or modes of administration. Understanding risk perceptions, as they vary across product and modes of administration is critical as they have differential impacts on the body. The pharmacological effects of cannabis vary depending on the concentrations of its primary active ingredient, THC (tetrahydrocannabinol, Δ^9^-THC), as well as whether products are smoked, vaped, or ingested orally or otherwise [20]. The THC content of cannabis has increased significantly over the past three decades in North American markets in its dried herbal form and has reached up to 80 to 90% in high potency products such as oils or waxes [21–23]. In general, frequent use of products containing higher THC concentrations significantly increases the risk of psychotic disorders, paranoia, marked effects on memory, and dependence [24–28]. Synthetic cannabis is the term given to mixtures of synthetic compounds marketed as “herbal” mixtures (e.g., Spice, K2m, Mamba, Afghan Incense) that activate cannabinoid receptors. They are the largest, fastest-growing, and most diversified new psychoactive substances which have been observed to precipitate acute psychotic symptoms, fatal poisonings, acute myocardial infarction, delusions, renal dysfunction, among other effects [29–31]. Synthetic cannabinoid use among youth is an emerging phenomenon in North America, approximately 3% of young Canadians in Grades 7 to 12 have reported its use, and 12% of their American counterparts have reported the same; the Canadian Centre for Substance Abuse identified this as a substance of concern as early as 2014 [2, 32, 33].

According to decades of substance use research, increased perceived riskiness and health knowledge influences rates of substance use and are generally associated with reductions in use initiation, increased engagement in health protective behaviors, and long-term abstinence [34–39]. Individuals who perceive a substance such as cannabis to be high risk are generally less likely to use it, and vice versa [34]. When young people are made aware that use behaviors are not normative and their misperceptions about how many of their peers actually use substances are corrected, substance use behaviors tend to decrease [40, 41]. However, existing work from US contexts that have legalized medical and/or nonmedical cannabis are less clear, and the relationship between perceived risk, health knowledge, and use behaviors remains poorly understood with respect to cannabis [42, 43].

Young people access health information from a variety of channels. Of these, online spaces represent credible sources of information for youth and young adults [44, 45]. A study by Lamy and colleagues demonstrates how social media platforms like Twitter are able to disseminate information about cannabis that describes it as “pure,” “clean,” and a “natural medicine ”[46]. Ouellette and colleagues describe how YouTube users are spreading information about cooking with cannabis, receiving collectively 15.6 million views though little is currently known regarding the basic effects these preparations may have or about users’ habits of ingesting edibles [47]. Work by Ramo and colleagues identified more than 700 smartphone apps related to cannabis and characterized them as likely vehicles for cannabis-related information and misinformation [48]. Overall, however, there is little population-level data on sources of health information on cannabis among young people. This is important given that Health Canada is reportedly spending $100M on cannabis education over 6 years and should consider competing sources of information known to be available that may dampen education efforts [49].

The present study examined young Canadians’ awareness of negative health effects, a proxy for knowledge, associated with cannabis use, perceptions of risk and harm associated with cannabis use, perceptions of relative risk to other substances or forms of administration (e.g., smoking, vaping, edibles), how these vary by use intensity (e.g., occasional vs. daily use), and how those perceptions were influenced by reported exposure to public health education around cannabis use.

## Methods

### Design

An online cross-sectional survey was conducted from October 10 to October 24, 2017. The inclusion criteria were individuals aged 16 to 30 years with a Canadian IP address and included cannabis users and non-users. Recruitment occurred by e-mail through Léger’s consumer panel for web surveys consisting of approximately 400,000 active members; half of them sampled using probability-based methods using the Canadian Census, along with other non-probability based methods, including commercial surveys [50]. Respondents aged 16 to 30 were recruited across Canada directly with the exception of youth in Quebec where youth aged 16 and 17 were recruited through their parents; parental consent was obtained prior to Quebec youth accessing the survey. Respondents received remuneration from Léger in accordance with their usual incentive structure. All of the data provided by respondents were anonymous, and information was kept confidential. In all cases, respondents were provided with information about the study and asked to provide consent before participating. They were reassured their anonymity again after providing consent and proceeded to the survey. The study was reviewed by and received ethics clearance from the Office of Research Ethics at the University of Waterloo (ORE# 22392).

### Measures

Sociodemographic variables: sex (male, female), age, ethnicity (white, non-white), cannabis use status (never use, recent use—use in the past 12 months, current use—use in the past 30 days) were recorded in the survey. Cannabis use status measures were used and adapted from the Canadian Student Tobacco, Alcohol and Drugs Survey (CSTADS), “Have you ever tried marijuana?” and a new item, “When was the last time you used marijuana” with options “More than 12 months ago,” “More than 3 months to 12 months ago,” “Within the last month”. Cannabis forms were asked about with the item, “In the past 12 months, did you used marijuana in any of the following ways: 1) Smoked dried herb/flower/leaf, 2) Vaporized dried flower/leaf 3) Vaporized liquid in an e-cigarette, 4) Mixed with or rolled in tobacco (e.g., blunt), 5) Hashish, 6) Hash oil, 7) Concentrate, 8) Edibles (e.g., cookies), 9) Liquid (e.g., cola/tea), 10) Tinctures (e.g., concentrated amounts ingested orally or taken under the tongue), 11) Topical ointments, 12) Fresh flower/leaf” with answer options “No, I have never done this,” “Yes, but not in the past 12 months,” “Yes, in the past 12 months,” “Don’t know,” “Refuse to answer” [51].

Exposure to education on risks related to cannabis and channels of exposure were assessed with the questions, “Have you seen any education campaigns or public health messages warning about the risks of marijuana use in the past 12 months?” followed up by, “Where have you seen education campaigns or public health messages about marijuana in the past 12 months?” with answer options outlined in Table 2. A binary variable was created to determine exposure to education on risks where 0 referred to “No exposure,” and 1 to “Exposed” where respondents had indicated that they had been exposed to any of the different channels asked about.

Awareness of negative health effects was assessed with the question, “In your opinion, what are the most important negative health effects from marijuana use? Please list up to five.” These open-ended responses were coded and classified into three categories: *Physiological effects*, *psychological effects*, and *social effects*.

Perceptions of risk and harm were assessed using four measures. General perceptions of harmfulness: (1) *Perceived likelihood of addiction*: “How likely is someone to become addicted to smoking marijuana?” with answer options, “0 = Very unlikely,” “0 = Somewhat unlikely,” “0 = Neither likely nor unlikely,” “1 = Somewhat likely,” “1 = Very Likely,” “0 = Don’t Know,” and (2) *Mental health harm:* “In general, do people risk harming their mental health when they use marijuana on a regular basis, for non-medical reasons?”, with answer options, “0 = No risk,” “1 = Slight risk,” “1 = Moderate risk,” “1 = Great risk,” “0 = I don’t know”. Perceptions of harm to self only asked of past 3-month users: (3) *Worry for future health:* “Are you worried that using marijuana will damage your health in the future?” with answer options, “0 = Not at all worried,” “1 = Yes, a little worried,” “1 = Yes, moderately worried,” “1 = Yes, very worried,” and “0 = Don’t know”. (4) *Perceived addiction:* “Do you consider yourself addicted to marijuana?” with answer options, “0 = Not at all,” “1 = Yes, a little addicted,” “1 = Yes, very addicted,” “0 = Don’t know”. Binary variables were created based on respondents’ answers for each measure (1) 0 = not likely, 1 = likely; (2) 0 = not worried, 1 = worried; (3) 0 = no risk, 1 = risk; and (4) 0 = not addicted, 1 = addicted.

Perceptions of risk depending on form of use were adapted from the 2015 European School Survey Project on Alcohol and Other Drugs (ESPAD) [52]. Participants were asked how much they risked harming themselves by the occasional or daily use of the following: smoking cannabis, vaporizing cannabis, eating or drinking cannabis, using high potency cannabis extracts, and using synthetic cannabis with answer options, “0 = No risk,” “1 = Slight risk,” “1 = Moderate risk,” “1 = Great risk,” “0 = I don’t know”. Binary variables were created based on respondents’ answers (0 = no risk, 1 = risk) for each cannabis form and frequency of use asked about. Responses were filtered by whether participants used cannabis in those ways.

Data integrity was assessed using two questions: “What is the current month?” and “One last question, did you feel you were able to provide honest answers about your marijuana use during the survey?” If respondents selected the wrong month or respondent felt unable to provide honest answers for “all questions,” they were excluded from the analytic sample.

### Analysis

All analyses were conducted using SPSS (Version 25.0, Armonk, NY: IBM Corp.). Survey weights were generated based on preliminary postcensal population estimates from Statistics Canada [53, 54]. Logistic regression models tested whether cannabis use was associated with perceived likelihood of addiction to smoking cannabis, worry for future health, mental health harm, and perceived addiction. Logistic regressions also examined whether different forms and modes of administration or use of different forms of cannabis and synthetic cannabinoids were associated with risk (physical or in other ways). All of the logistic regressions were adjusted for age, sex, ethnicity, cannabis use status, and exposure to cannabis risk education. Unless otherwise noted, all analyses are based on weighted data.

## Results

Table 1 displays the sample characteristics. Overall, 1045 respondents completed the survey. However, due to missing data on core measures of cannabis use and/or failed data integrity questions, a final sample of 870 youth and young adults was analyzed. A total of 1045 respondents completed the survey; however, the final analytic sample was 870 as the rest were excluded from analysis due to completing survey from a mobile device instead of a desktop computer (28), missing data on key measures including cannabis use status (8), and/or failed data integrity questions; 62 records were deleted due to incorrectly identifying the current month and 77 respondents reported being unable to provide honest answers to all of the survey questions.

**Table 1:** Sample characteristics

		Unweighted % (***N***) ***N*** = 870	Weighted % (***N***) ***N*** = 867
Sex	Female	52.1 (453)	49.2 (427)
	Male	47.9 (417)	50.8 (441)
Age (years)	16–18	25.2 (219)	17.0 (148)
	19–24	30.7 (267)	40.3 (350)
	25–30	44.1 (384)	42.7 (370)
Ethnicity	White	64.5 (561)	64.6 (560)
	Non-white	35.5 (309)	35.4 (307)
Cannabis use status	Never use	41.5 (361)	37.4 (325)
	Ever use, not in past 30 days	36.0 (313)	38.7 (336)
	Current use, within past 30 days	22.5 (196)	23.8 (207)
Exposed to education on risks associated with cannabis		31.8 (277)	32.8 (284)

### Exposure to education or public health messaging about cannabis use

Less than one-third of respondents (31.8%) reported encountering public health messages about cannabis in the past year. Table 2 displays the different locations where reported encounters took place. The most common locations reported were online (16.7%), on television or radio (15.2%), and in school (12.5%).

**Table 2:** Location where education campaigns or public health messages about cannabis were encountered by participants in the past year (*n* = 870)

Location	% (***n***)
On websites or social media like Facebook, Twitter, YouTube, Instagram, or Snapchat	16.7 (145)
On television or radio	15.2 (132)
In school	12.5 (109)
On billboards and posters	6.8 (59)
In print newspapers or magazines	5.4 (47)
At a pharmacy	4.1 (36)
At work	3.9 (34)
In e-mail or text messages	2.1 (18)
In bars or pubs	2.1 (18)
Taxis or buses/public transit	2.1 (18)
At events like fairs, markets, festivals, sporting events, or music concerts	2.0 (17)
In flyers	1.6 (14)
In shops/stores that sell marijuana	1.5 (13)
At the movies	1.4 (12)
At kiosks or temporary sales locations (in shopping centers, parked in the street, other places, but not at specific events)	1.1 (10)
Outside shops/stores that sell marijuana	1.0 (9)
Do not know	0.8 (7)

### Awareness of negative health effects

Table 3 summarizes the “most important” negative health effects associated with cannabis use reported by respondents. Overall, 78.0% of all respondents cited at least one physical concern, 43.0% cited at least one psychological concern, while 4.5% cited at least one social concern.

**Table 3:** Frequencies of “most important” negative health effects associated with cannabis use reported by Canadian youth and young adults (*N* = 870)

	%	(***N***)
**Physical concerns**		
Decreased brain function, cognitive abilities	24.7	(215)
Respiratory function	23.8	(207)
Addiction	16.4	(143)
Carcinogenic	9.7	(84)
Harmful effects from smoking	7.2	(63)
Hunger/“munchies”	6.7	(58)
Drowsiness	6.1	(53)
General detriment to health	5.4	(47)
Issues with circulation, heart palpitations	5.3	(46)
Dry mouth	3.6	(31)
Youth health, developmental concerns	3.0	(26)
Bad/“gross” smell	2.6	(23)
Drug interactions, concerns about tainted product	2.1	(18)
Obesity	2.0	(17)
Dizziness	1.8	(16)
Nausea	1.6	(14)
Red eyes	1.5	(13)
Vision problems	1.4	(12)
Sexual health	1.4	(12)
Death	1.1	(10)
**Psychological Concerns**		
Reaction time related to driving or other activities	9.3	(81)
Mental health issues	9.3	(81)
Judgment/Inhibition issues	8.5	(74)
Memory loss	8.4	(73)
Loss of motivation	7.5	(65)
Anxiety/panic	5.5	(48)
Depression	3.7	(32)
Hallucinations	3.7	(32)
Loss of concentration	3.7	(32)
Paranoia	3.4	(30)
Being high	1.6	(14)
Schizophrenia	1.1	(10)
Psychosis	1.1	(10)
**Social concerns**		
Gateway drug	1.7	(15)
Behavioural changes	1.5	(13)
Money problems	1.0	(9)
**Do not know/refused**		
Do not know	10.0	(87)
Refused	1.7	(15)

*Concerns accounting for < 1% of overall responses are omitted here

Among physical concerns, effects on the brain and respiratory function were the most common responses. *Never* and *ever* users were more likely to report physical concerns related to cannabis use than *current* users (AOR = 1.89, 95% CI 1.26–2.85, *p =* 0.002, AOR = 1.57, 95% CI 1.07–2.31, *p =* 0.002, respectively). Furthermore, respondents that reported having encountered public health messaging were more likely to report physical concerns than those that had not (AOR = 2.31, 95% CI 1.60–3.34, *p* < 0.001).

Deficits in reaction time in relation to driving among other activities, general mental health issues, and memory loss were the most frequently cited psychological concerns. Older respondents aged 19–24 years and those aged 25–30 were at a greater likelihood of reporting psychological concerns than youth aged 16–18 years (AOR = 1.58, 95% CI 1.09–2.30, *p =* 0.015, AOR = 1.49, 95% CI 1.04–2.14, *p =* 0.032, respectively). Encountering public health messaging was also associated with a greater likelihood of reporting a psychological concern (AOR = 2.26, 95% CI 1.72–2.97, *p* < 0.001).

Respondents cited that cannabis may be a gateway drug and were concerned about behavioral changes as social concerns.

### Perceptions of risk and harm

Table 4 presents respondents’ perceptions of health harms associated with cannabis use, and Table 5 displays related logistic regression analyses examining each risk.

**Table 4:** Perceptions of health effects associated with cannabis use (*N* = 870)

		Very unlikely	Somewhat unlikely	Neither likely nor unlikely	Somewhat likely	Very likely	Do not know
Beliefs, others	Likely someone may become addicted from smoking	14.7 (128)	16.0 (139)	17.0 (148)	27.6 (240)	16.6 (144)	7.9 (69)
		**No risk**	**Slight risk**	**Moderate risk**	**Great risk**	**Do not know**	
	Risk of harming mental health	10.9 (95)	32.2 (280)	27.2 (237)	21.8 (190)	7.8 (68)	
		**Not at all worried**	**A little worried**	**Moderately worried**	**Very worried**	**Do not know**	
Beliefs, self	Worry about damage to future health*	51.3 (137)	34.1 (91)	9.0 (24)	2.6 (7)	3.0 (8)	
		**Not at all**	**Yes, a little addicted**	**Yes, very addicted**	**Do not know**		
	Perceived addiction*	76.0 (203)	20.2 (54)	2.6 (7)	1.1 (3)		

*Among past 3-month users (*n* = 267)

**Table 5:** Logistic regression analyses examining potential risks associated with cannabis use among Canadian youth and young adult cannabis users and non-users

Characteristics		Ref. Category	Likely someone may become addicted from smoking, general (***N*** = 870)		
			*p*	AOR	95% CI
**Age**	19–24	16–18	**0.001**	**0.51**	**0.35–0.76**
	25–30		**< 0.001**	**0.49**	**0.34–0.70**
	19–24	25–30	0.738	1.06	0.76 = 1.48
**Sex**	Male	Female	**0.013**	**0.69**	**0.52–0.93**
**Ethnicity**	White	Non-white	0.169	0.81	0.60–1.90
**Cannabis use status**	Ever use, not in past 30 days	Never use	**< 0.001**	**0.39**	**0.28–0.55**
	Current use, within past 30 days		**< 0.001**	**0.35**	**0.24–0.52**
	Ever use, not in past 30 days	Current use, within past 30 days	0.597	1.11	0.75–1.64
**Exposure to education**	Exposed	Not exposed	0.097	1.29	0.96–1.76
			**Risk of harming mental health, general (*N*= 870)**		
**Age**	19–24	16–18	0.533	0.85	0.51–1.42
	25–30		0.829	0.95	0.59–1.54
	19–24	25–30	0.599	0.90	0.60–1.35
**Sex**	Male	Female	0.807	0.96	0.67–1.36
**Ethnicity**	White	Non-white	0.945	1.01	0.70–1.48
**Cannabis use status**	Ever use, not in past 30 days	Never use	**0.001**	**0.48**	**0.31–0.74**
	Current use, within past 30 days		**< 0.001**	**0.35**	**0.22–0.56**
	Ever use, not in past 30 days	Current use, within past 30 days	0.155	1.36	0.89–2.08
**Exposure to education**	Exposed	Not exposed	**< 0.001**	**2.28**	**1.50–3.48**
			Worry about damage to future health, self (*n*= 267)*		
**Age**	19–24	16–18	0.080	0.51	0.24–1.08
	25–30		0.492	0.78	0.38–1.59
	19–24	25–30	0.140	0.66	0.38–1.15
**Sex**	Male	Female	0.572	1.16	0.70–1.91
**Ethnicity**	White	Non-white	0.349	0.78	0.45–1.32
**Cannabis use status**	Current use, within past 30 days	Ever use, not in past 30 days	0.721	1.11	0.63–1.95
**Exposure to education**	Exposed	Not exposed	**0.010**	**1.99**	**1.18–3.37**
			Perceived addiction, self (*n*= 267)*		
**Age**	19–24	16-18	**0.048**	**0.41**	**0.17–0.99**
	25–30		0.220	0.61	0.27–1.35
	19–24	25–30	0.287	0.68	0.34–1.38
**Sex**	Male	Female	0.068	1.78	0.96–3.30
**Ethnicity**	White	Non-white	0.770	0.91	0.47–1.74
**Cannabis use status**	Current use, within past 30 days	Ever use, not in past 30 days	**0.003**	**3.96**	**1.60–9.78**
**Exposure to education**	Exposed	Not exposed	0.125	1.62	0.88-2.99

*Among past 3-month users

#### Perceived likelihood of addiction from smoking cannabis

As indicated in Table 4, perceptions of cannabis addiction varied widely across the sample. Logistic regression analyses shown in Table 5 indicated that young people aged 19–24 and 25–30 years were less likely to report that someone may become addicted to cannabis than youth aged 16–18 years (AOR = 0.51, 95% CI 0.35–0.76, *p* = 0.001 and AOR = 0.49, 95% CI 0.34–0.70, *p* < 0.001 respectively). Compared to female respondents, males perceived cannabis as less addictive (AOR = 0.69, 95% CI 0.52–0.93, *p* = 0.013). Compared to *never* cannabis users, *ever* and *current* cannabis users were also less likely to report that cannabis users would become addicted (AOR = 0.39 95% CI 0.28–0.55, *p* < 0.001; AOR = 0.35 95% CI 0.24–0.52, *p* < 0.001 respectively). No other significant differences were observed.

#### Mental health harm

One in ten (10.9%) respondents reported that they perceived “no risk” of harming mental health as a result of using cannabis. *Ever* and *current* cannabis users had lower odds of reporting that individuals that use cannabis risk harming their mental health than those who reported that they had *never* used cannabis (AOR = 0.48, 95% CI 0.31–0.74, *p* = 0.001; AOR = 0.35, 95% CI 0.22–0.56, *p* < 0.001, respectively). Furthermore, those who reported having been exposed to some form of public health messaging regarding risks associated with cannabis were more likely to report that users risk harming their mental health (AOR = 2.28, 95% CI 1.50–3.48, *p* < 0.001).

#### Worry for future health

Among those who reported using cannabis in the past 3 months, about half reported being worried about potential damage to future health (51.3%). There were no significant differences between “daily,” “weekly,” “monthly,” or “less than monthly” cannabis users in responding affirmatively to worrying about damaging future health as a result of their use. However, those that reported seeing public health messaging were more likely to report this type of worry (AOR = 1.99, 95% CI 1.18–3.37, *p* = 0.010).

#### Self-perceived addiction

More than 1 in 5 (22.8%) of respondents who reported using cannabis within the past 3 months reported being “a little” or “very” addicted. Those that reported using in the past 30 days were more likely to consider themselves addicted than those that reported not having used cannabis in the past 30 days (AOR = 3.96, 95% CI 1.60–9.78, *p* = 0.003). Among past 3-month users, respondents that reported “weekly,” “monthly,” or “less than monthly” use were significantly less likely to report perceiving themselves as addicted to cannabis than “daily” users (AOR = 0.10, 95% CI 0.04–0.24, *p* < 0.001; AOR = 0.09, 95% CI 0.03–0.25, *p* < 0.001; AOR = 0.04, 95% CI 0.01–0.11, *p* < 0.001, respectively).

### Perceptions of risk of harm (physical or in other ways) to other forms of administration

**Fig. 1:**
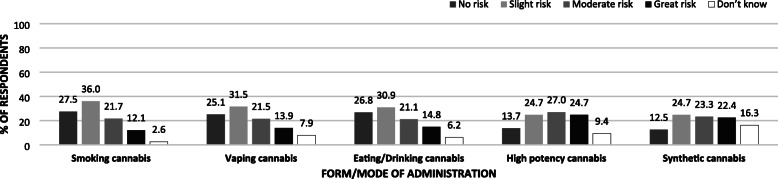
Perceptions of level of health risks associated with occasional cannabis use by cannabis form/mode of administration (*N* = 870)

**Fig. 2:**
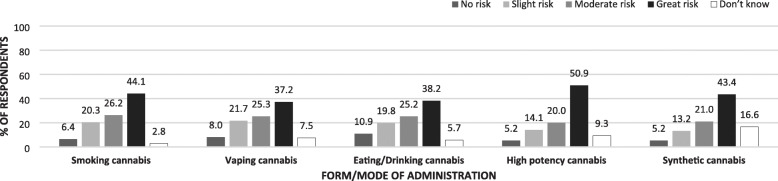
Perceptions of level of health risks associated with daily cannabis use with cannabis form/mode of administration (*N* = 870)

Figures 1 and 2 display respondents’ perceptions of health risk associated with different forms of cannabis use. Overall, daily use of any form of cannabis was perceived to carry greater risk than occasional use. Half (50.9%) of all respondents reported that daily use of high potency cannabis carried “great risk” of harm, physically or in other ways, and approximately 4 in 10 reported the same for smoking (44.1%), synthetic cannabis use (43.4%), eating or drinking cannabis (38.2%), and vaping cannabis (37.2%). In contrast, less than 1 in 4 thought the same regarding the occasional use of any form of cannabis use.

Table 6 displays the results of logistic regression analyses examining correlates of perceived harm associated with daily cannabis use in its different forms. Similar patterns emerged across forms; cannabis users consistently display lower odds of perceiving harm from the daily use of different forms of cannabis than respondents that report never having used cannabis. However, a gradient among cannabis users is evident; those that reported having used, but not in the past 30 days, are more likely to report risks of harm from daily cannabis use than those that report having used in the past 30 days. Finally, reporting exposure to cannabis risk public health messaging was associated with increased likelihood of perceiving harm from daily use of different forms of cannabis with the exception of edible or drinkable forms of administration. Similar patterns emerge when respondents were asked about occasional use of the different cannabis forms. Table S3 in the Supplementary File also displays logistic regression analyses examining correlated of perceived harm associated with different intensities of use (occasional and daily use) and different forms of cannabis; however, it is filtered further by whether participants used cannabis in each way. Across the different modes of cannabis self-administration, participants that indicated using each particular form were less likely to perceive risk of harm than those that did not use the particular form (*p* < 0.001).

**Table 6:** Logistic regression analyses examining risks of harm (physical or in other ways) perceived by cannabis forms and frequency of use among Canadian youth and young adult cannabis users and non-users (*N* = 867)

Characteristics		Ref. category	Smoke cannabis daily			Vape cannabis daily			Eat/drink cannabis daily			Use high potency extracts daily			Use synthetic cannabis daily		
			*p*	AOR	95% CI	*p*	AOR	95% CI	*p*	AOR	95% CI	*p*	AOR	95% CI	*p*	AOR	95% CI
**Cannabis use status**	Current use, use in past 30 days	Never use	**< 0.001**	**0.21**	**0.11–0.40**	**< 0.001**	**0.40**	**0.25–0.64**	**< 0.001**	**0.24**	**0.15–0.39**	**< 0.001**	**0.32**	**0.20–0.52**	**0.008**	**0.58**	**0.39–0.87**
	Ever use, not in the past 30 days		**0.013**	**0.45**	**0.23–0.84**	0.343	0.80	0.51–1.27	**0.002**	**0.48**	**0.30–0.77**	0.056	0.63	0.39–1.01	0.446	0.86	0.59–1.26
	Ever use, not in past 30 days	Current use, use in past 30 days	**0.003**	**2.12**	**1.30–3.47**	**0.001**	**2.01**	**1.31–3.08**	**0.001**	**2.01**	**1.34–2.99**	**0.002**	**1.96**	**1.28–3.01**	**0.041**	**1.49**	**1.02–2.18**
**Exposure to education**	Exposed	Not exposed	**0.007**	**2.09**	**1.22–3.57**	**< 0.001**	**2.27**	**1.48–3.50**	0.054	1.47	0.99–2.16	**0.006**	**1.80**	**1.18–2.73**	**0.028**	**1.46**	**1.04–2.05**

## Discussion

Canadian youth and young adults reported being aware of a number of important health effects associated with cannabis use, as well as a number of channels where they have been receptive to public health messaging or educational information. Approximately a third of young Canadians reported being exposed to public health messages on cannabis, with digital media, television and radio being the most reported channels of exposure in our study. The findings reinforce the importance of digital media in reaching young people. Virtually all (95%) Canadians aged 18 to 24 years use Facebook and nearly all (90%) use YouTube, with a majority being active monthly users of these platforms [55]. Exposure to education campaigns may have increased immediately following the current study, given the range of campaigns implemented in the lead up to cannabis legalization, in October 2018. For example, Public Safety Canada launched a “Don’t Drive High” campaign the following month and reported that 62% of Canadians aged 16 to 24 years recalled the message post-campaign [56]. The Government of Canada has also engaged a number of partner organizations such as Drug Free Kids Canada, Mothers Against Drunk Driving, the Canadian Hockey League, and the Centre of Addictions and Mental Health, and made funding available for research and outreach in the area to target dissemination efforts of cannabis and health information [56]. Future studies should examine the population-level reach of these campaigns.

Overall, young people reported a wide range of beliefs about the health effects of cannabis use. Indeed, responses were equally distributed across the scale of likelihood of addiction and risks of mental health. For example, similar proportions reported that cannabis addiction from smoking was “very unlikely” and “very likely”. This pattern of results reflects the wide diversity of opinions about addiction to cannabis: as the Canadian Task Force on Cannabis Legalization and Regulation noted, cannabis is one of the few substances for which substantial proportions of the public both underestimate and overestimate the health effects [57]. Interestingly, approximately one-quarter of past 3-month cannabis users reported they were at least “a little” addicted to cannabis, and this proportion increased with more frequent use. While perceptions of addiction among users are lower than for other substances, such as tobacco smoking, the findings are consistent with studies suggesting problematic use in a certain minority of users [18, 58].

Future studies should prioritize perceptions of cannabis and mental health. The current study indicates a wide range of beliefs about the effects of cannabis on mental health. Indeed, many young Canadians perceive cannabis to have a net overall benefit on public health believing that it may be used to treat mental disorders, cure cancer, and a strong notion that cannabis does not have long-term health effects [12]. Future work should discriminate between different aspects of mental health, such as depression and anxiety versus psychosis, for which there is a strong evidence base on negative impact [17]. Investigations should also examine how perceived benefits related to cannabis use are informed by young Canadians’ lived reality to craft better evidence-based and focussed public health programming.

The findings also suggest that cannabis users may be demonstrating a form of “optimism” bias: worry about health effects and perceptions of addiction appeared to be lower when they were personalized to users rather than asked in the general form. This is similar to optimism bias exhibited by smokers, who often underestimate their own risks relative to others [59].

The current study is among the first to examine whether perceptions of risk differ across product types and modes of administration. Daily use in all of its forms was consistently perceived to be more risky than occasional cannabis use, as expected. In terms of modes of administration, smoked, vaped, and edible cannabis products were perceived to have similar risk, whereas high potency products and synthetic cannabis products were perceived as somewhat riskier. However, similar proportions of young people perceived daily use of synthetic cannabis and daily cannabis smoking to be “high risk”. This is a concern given the serious acute health effects of synthetic cannabis use, particularly given their “legal” status in many jurisdictions and widespread consumer confusion about these products [29–31, 60, 61]. Focused attention should be given to highlighting and detailing the risks associated with synthetic cannabinoid that dwarf existing known risks associated with combusted cannabis. The findings suggest that public education campaigns implemented as part of cannabis legalization in Canada should help consumers to identify and understand the unique health effects of synthetic cannabis products.

### Strengths and limitations

The sample was recruited from a commercial sample that used probability and non-probability-based recruitment methods, thus, our findings may not be fully generalizable to Canadian young people. Nevertheless, a broad and diverse sample with similar patterns of cannabis use and sociodemographic characteristics as the 2017 Canadian Cannabis Survey was recruited [62]. The current sample surveyed aged 16 to 30 years exclusively. While this age group has the highest rates of use in Canada, it is unclear to what extent the current findings are representative of older adults. It should be noted that directionality regarding beliefs and exposure or noticing public health messaging is unclear and an opportunity for future research. Considerable strengths of the study include the use of existing international tools that allow for comparability to countries participating in the ESPAD, open-ended fields that allowed respondents to freely communicate the extent of their cannabis health knowledge, and this study expands the limited literature on young Canadians’ cannabis health knowledge and risk perceptions.

## Conclusion

The current study is among the first to measure cannabis health knowledge and perceptions of risks among Canadian youth in the time immediately preceding legalization; it may serve as a reference point for future studies examining changes in cannabis literacy. The findings complement and add to the very limited literature specific to young Canadians’ cannabis health knowledge and ability to evaluate associated risks [11–13]. The findings indicate that a minority of young people recall seeing or hearing public education on cannabis products over the past year, although those that do report greater perceptions of risk. More generally, the study highlights the wide discrepancy of views about the potential health effects and addictive potential of cannabis. Legalization of non-medical cannabis provides an opportunity—and indeed a mandate—to enhance public education on the health effects of cannabis. Helping consumers to understanding different modes of administration and the distinct risk profile of synthetic cannabis should be an important component of public education campaigns.

## Supplementary information

**Additional file 1.** Supplemental file.

## Data Availability

Not available for public dissemination.

## Abbreviations

CCS: Canadian Cannabis Survey
CSTADS: 2016-17 Canadian Student Tobacco, Alcohol and Drugs Survey
ESPAD: European School Survey Project on Alcohol and Other Drugs
THC: Tetrahydrocannabinol
