# Supplementary material for: Cannabis health knowledge and risk perceptions among Canadian youth and young adults
Source: Harm Reduct J. 2020 Aug 3;17:54. doi: 10.1186/s12954-020-00397-w (PMC7398180; doi:10.1186/s12954-020-00397-w)
Supplement: Supplementary file 1 — Additional file 1. Supplemental file. [file 12954_2020_397_MOESM1_ESM.docx]

**SUPPLEMENTARY FILE**

Table S1 - Risk perceptions of OCCASIONAL USE reported by past 12-month cannabis users by cannabis form (n=322)

| **RISK** |  | **0=No Risk** | **1=Risk** | | | | |
| --- | --- | --- | --- | --- | --- | --- | --- |
|  |  | **No risk**  **%(n)** | **Slight risk** | **Moderate risk** | **Great risk** | **Don’t know** | **Total**  **% (n)** |
| Smoking | Smoked (n=165) | 12.3 (107) | 45 | 12 | 0 | 1 | 6.7 (58) |
| Edibles | Edibles (n=110) | 8.0 (70) | 25 | 10 | 3 | 2 | 4.6 (40) |
|  | Liquid (e.g. cola/tea) (n=25) | 0.9 (8) | 9 | 4 | 3 | 1 | 2.0 (17) |
|  | Tinctures (n=26) | 1.6 (14) | 7 | 3 | 1 | 1 | 1.4 (12) |
|  | Fresh flower (n=14) | 0.8 (7) | 5 | 1 | 1 | 0 | 0.8 (7) |
| Vaping | Vaporized (dried herb) (n=91) | 6.4 (56) | 25 | 8 | 0 | 2 | 4.0 (35) |
|  | Vaporized (liquid) (n=62) | 4.3 (37) | 11 | 12 | 61 | 2 | 9.9 (86) |
| High Potency Product | Hashish (n=69) | 4.3 (37) | 17 | 9 | 3 | 2 | 3.6 (31) |
|  | Hash Oil (n=43) | 2.4 (21) | 12 | 5 | 3 | 2 | 2.5 (22) |
|  | Concentrate (e.g., BHO, shatter, budder, wax etc.) (n=61) | 2.4 (21) | 21 | 10 | 4 | 5 | 4.6 (40) |

Table S2 - Risk perceptions of DAILY USE reported by past-12 month cannabis users by cannabis form (n=322)

| **RISK** |  | **0=No Risk** | **1= Risk** | | | | |
| --- | --- | --- | --- | --- | --- | --- | --- |
|  |  | **No risk** | **Slight risk** | **Moderate risk** | **Great risk** | **Don’t know** | **Total** |
| Smoking | Smoked (n=165) | 3.3 (29) | 69 | 47 | 20 | 1 | 15.7 (137) |
| Edibles | Edibles (n=110) | 4.4 (38) | 36 | 24 | 9 | 3 | 8.3 (72) |
|  | Liquid (e.g. cola/tea) (n=25) | 0.6 (5) | 7 | 5 | 7 | 0 | 2.2 (19) |
|  | Tinctures (n=26) | 1.3 (11) | 10 | 3 | 1 | 0 | 1.6 (14) |
|  | Fresh flower (n=14) | 0.2 (2) | 9 | 2 | 1 | 0 | 1.4 (12) |
| Vaping | Vaporized (dried herb) (n=91) | 2.5 (22) | 39 | 19 | 10 | 2 | 8.0 (70) |
|  | Vaporized (liquid) (n=62) | 1.5 (13) | 19 | 16 | 12 | 1 | 5.5 (48) |
| High Potency Product | Hashish (n=69) | 1.6 (14) | 24 | 16 | 13 | 2 | 6.3 (55) |
|  | Hash Oil (n=43) | 1.3 (11) | 17 | 5 | 7 | 2 | 3.6 (31) |
|  | Concentrate (e.g., BHO, shatter, budder, wax etc.) (n=61) | 1.4 (12) | 16 | 12 | 16 | 6 | 5.7 (50) |

Table S3 - Logistic regression analyses examining risks of harm (physical or in other ways) perceived by cannabis forms and frequency of use among Canadian youth and young adult cannabis users and non-users (N=867).

| Characteristics | | *Ref. Category* | **Smoking Cannabis Occasionally** | | | **Smoking Cannabis Daily** | | |
| --- | --- | --- | --- | --- | --- | --- | --- | --- |
|  |  |  | *p* | AOR | 95%CI | *p* | AOR | 95%CI |
| **Age** | 19-24 | 16-18 | **<0.001** | **0.45** | **0.30-0.70** | 0.509 | 0.80 | 0.40-1.57 |
|  | 25-30 |  | **0.003** | **0.54** | **0.36-0.81** | 0.122 | 0.61 | 0.33-1.14 |
|  | 19-24 | 25-30 | 0.300 | 0.84 | 0.61-1.17 | 0.298 | 1.31 | 0.79-2.15 |
| **Sex** | Male | Female | 0.677 | 0.94 | 0.70-1.26 | 0.262 | 1.29 | 0.83-2.02 |
| **Cannabis use** | Smokes | Does not smoke | **<0.001** | **0.156** | **0.11-0.22** | **<0.001** | **0.30** | **0.19-0.48** |
| **Ethnicity** | White | Non-white | **<0.001** | **0.51** | **0.37-0.70** | **0.080** | **0.64** | **0.381.06** |
| **Exposure to education** | Exposed | Not Exposed | **0.010** | **1.53** | **1.11-2.12** | **0.006** | **2.13** | **1.25-3.65** |
|  |  |  | **Vaping Cannabis Occasionally** | | | **Vaping Cannabis Daily** | | |
|  |  |  | *p* | AOR | 95%CI | *p* | AOR | 95%CI |
| **Age** | 19-24 | 16-18 | **0.002** | **0.54** | **0.37-0.80** | 0.139 | 0.67 | 0.40-1.14 |
|  | 25-30 |  | **0.002** | **0.56** | **0.38-0.81** | 0.082 | 0.64 | 0.39-1.06 |
|  | 19-24 | 25-30 | 0.858 | 0.97 | 0.72-1.32 | 0.828 | 1.05 | 0.70-1.6 |
| **Sex** | Male | Female | 0.448 | 0.90 | 0.68-1.18 | 0.347 | 1.19 | 0.83-1.70 |
| **Cannabis use** | Vapes | Does not vape | **<0.001** | **0.28** | **0.19-2.19** | **<0.001** | **0.42** | **0.26-0.67** |
| **Ethnicity** | White | Non-white | **0.001** | **0.60** | **0.44-0.80** | 0.208 | 0.78 | 0.53-1.14 |
| **Exposure to education** | Exposed | Not Exposed | **0.002** | **1.62** | **1.20-2.20** | **<0.001** | **2.28** | **1.48-3.52** |
|  |  |  | **Eating/Drinking Cannabis Occasionally** | | | **Eating/Drinking Cannabis**  **Daily** | | |
|  |  |  | *p* | AOR | 95%CI | *p* | AOR | 95%CI |
| **Age** | 19-24 | 16-18 | **0.003** | **0.54** | **0.36-0.81** | 0.515 | 0.84 | 0.50-1.42 |
|  | 25-30 |  | **0.002** | **0.55** | **0.38-0.80** | **0.044** | **0.61** | **0.38-0.99** |
|  | 19-24 | 25-30 | 0.929 | 0.99 | 0.72-1.35 | 0.115 | 1.38 | 0.93-2.06 |
| **Sex** | Male | Female | 0.479 | 0.90 | 0.68-1.20 | 0.891 | 0.98 | 0.69-1.39 |
| **Cannabis use** | Consumes edibles | Does not consume edibles | **<0.001** | **0.22** | **0.15-0.33** | **<0.001** | **0.26** | **0.17-0.39** |
| **Ethnicity** | White | Non-white | **<0.001** | **0.55** | **0.41-0.74** | 0.158 | 0.76 | 0.52-1.11 |
| **Exposure to education** | Exposed | Not Exposed | 0.066 | 1.33 | 0.98-1.80 | 0.062 | 0.26 | 0.17-0.39 |
|  |  |  | **Use High Potency Extracts Occasionally** | | | **Use High Potency Extracts**  **Daily** | | |
|  |  |  | *p* | AOR | 95%CI | *p* | AOR | 95%CI |
| **Age** | 19-24 | 16-18 | 0.231 | 0.77 | 0.50-1.18 | 0.392 | 0.80 | 0.48-1.33 |
|  | 25-30 |  | 0.343 | 0.82 | 0.55-1.23 | 0.612 | 0.88 | 0.54-1.43 |
|  | 19-24 | 25-30 | 0.710 | 0.94 | 0.67-1.32 | 0.641 | 0.91 | 0.61-1.36 |
| **Sex** | Male | Female | 0.689 | 1.06 | 0.79-1.44 | 0.953 | 0.99 | 0.69-1.42 |
| **Cannabis use** | Uses high potency extracts | Does not use high potency extracts | **<0.001** | **0.24** | **0.16-0.37** | **0.002** | **0.45** | **0.28-0.74** |
| **Ethnicity** | White | Non-white | 0.053 | 0.72 | 0.52-1.00 | 0.944 | 0.99 | 0.67-1.44 |
| **Exposure to education** | Exposed | Not Exposed | **0.003** | **1.68** | **1.20-2.36** | **0.009** | **1.74** | **1.15-2.64** |
